# Relaxing the independent censoring assumption in the Cox proportional hazards model using multiple imputation

**DOI:** 10.1002/sim.6274

**Published:** 2014-07-25

**Authors:** Dan Jackson, Ian R White, Shaun Seaman, Hannah Evans, Kathy Baisley, James Carpenter

**Affiliations:** 1 MRC Biostatistics Unit, Institute of Public HealthForvie Site, Robinson Way, Cambridge, CB2 0SR, U.K.; 2 Department of Medical Statistics, London School of Hygiene and Tropical MedicineKeppel Street, London, WC1E 7HT, U.K.; 3 MRC Clinical Trials UnitLondon, WC2B 6NH, U.K.

**Keywords:** bootstrapping, informative censoring, multiple imputation, Schoenfeld residuals, sensitivity analysis, survival analysis

## Abstract

The Cox proportional hazards model is frequently used in medical statistics. The standard methods for fitting this model rely on the assumption of independent censoring. Although this is sometimes plausible, we often wish to explore how robust our inferences are as this untestable assumption is relaxed. We describe how this can be carried out in a way that makes the assumptions accessible to all those involved in a research project. Estimation proceeds via multiple imputation, where censored failure times are imputed under user-specified departures from independent censoring. A novel aspect of our method is the use of bootstrapping to generate proper imputations from the Cox model. We illustrate our approach using data from an HIV-prevention trial and discuss how it can be readily adapted and applied in other settings. © 2014 The Authors. *Statistics in Medicine* published by John Wiley & Sons, Ltd.

## 1 Introduction

Models for survival analysis 1–4 are very commonly applied to time-to-event data in medical statistics. Typically, the analysis is complicated because the failure times are unobserved for a proportion of individuals; instead, we record the last time that they were under observation, known as the censoring time. This type of censoring is called right censoring and will occur if individuals are still at risk of failure at the scheduled end of the study, but often a non-trivial proportion of participants will be right censored before this time.

Standard software assumes independent censoring, conditional on the covariates in the analysis model. However, this assumption is untestable and will often be doubtful for individuals censored before the scheduled end of the study. One reason for this is because dropout is a common reason for censoring. Censoring might therefore be thought to be indicative that the participant is more likely to subsequently fail more quickly because, for example, dropout could be associated with a deterioration in health and hence also associated with failure. In the most extreme case, a participant could be lost to follow-up, and hence censored, because failure is about to occur. On the other hand, the event of being censored might be thought to have a protective effect, because participants could drop out because their condition has improved and so no longer require the support of the trial that they enrolled in. Censoring could therefore plausibly have either a protective or a harmful effect depending on the circumstances.

Rather than focus on the myriad of reasons why the assumption of independent censoring may be false, here we develop a procedure to quantify the sensitivity of the conclusions from fitted Cox proportional hazards models 5 where, for whatever reason, the independent censoring assumption is in doubt. The Cox model is the most commonly used model for survival analysis. This semi-parametric model allows inferences to be made concerning covariate effects without the added complication of modelling the baseline hazard function. This model provides our focus, but we will explain how to adapt our methods so that they can be used in conjunction with parametric proportional hazards models.

We model the association between the censoring and failure mechanisms in a simple and direct manner, motivated by the intuition that censoring is associated with a step change in the hazard of failure. This step change may increase or decrease this hazard, so that censoring may be associated with either a harmful effect or a protective effect. By modelling the association between censoring and failure in such a transparent way, investigators can have an informed discussion about the model's assumptions and the plausible range of the sensitivity parameter or parameters. Our model is also easily interpretable, which makes it relatively simple to translate our model into a wide variety of applications. We derive our model in the context of a more general framework below and describe how this more general framework could be implemented in the discussion, so that our approach could also be used in situations where a sudden change in the hazard at the time of censoring is less plausible.

Related work has been carried out by Siannis and colleagues 6–8 who propose local sensitivity analyses, where the implications of small associations between the failure and censoring mechanisms are assessed. Our approach allows global sensitivity analyses but makes use of more computationally intensive methods. Bivariate models 9 and shared parameter (or ‘frailty’) models 10 are alternatives for invoking an association between the censoring and failure mechanisms. Scharfstein and Robins 11 make this association explicit by modelling the censoring mechanism conditionally on the failure time, but we avoid modelling the censoring mechanism. Methods using Kaplan–Meier (product limit) methods have also been developed 12. Our approach is similar in many respects to the one proposed by Zhao *et al.* 13 who use Kaplan–Meier curves to impute data under informative censoring. However, Zhao *et al.* do not include covariates and only describe the use of a single sensitivity parameter. Here, we provide a unified modelling framework that can incorporate much more complicated informative censoring mechanisms.

We regard the censored observations as providing missing (unobserved) failure times and use multiple imputation 14, Chapter 9] to impute censored failure times. Our method for imputing missing failure times is similar to the one proposed by Faucett *et al.* 15, but we include parameters that describe the departure from independent censoring and propose a bootstrap approach 16 instead of Markov chain Monte Carlo. Hsu and Taylor 17 and Liu *et al.* 18 generate ‘imputing risk sets’ or ‘imputing pools’ for each censored observation, but our method imputes failure times for censored participants from the entire sample of observed failure times that are greater than their censoring times. In multiple imputation, we take both the uncertainty in the imputation model and the sampling variation into account when creating the imputed datasets. Here, we also impute missing data given a user-specified sensitivity parameter or parameters that quantify the departure from independent censoring. These sensitivity parameters are the step change parameters in our model, which the data provide no information about. The independent censoring assumption is equivalent to assuming that all sensitivity parameters are zero. The term ‘sensitivity analysis’ covers a wide range of strategies, but the approach adopted here is to explore the implications of a range of possible values of the sensitivity parameters. This approach for performing sensitivity analyses has been referred to as a ‘principled sensitivity analysis’ 19. The observed data that we impute the missing failure times conditionally on include the censoring times, so that the imputed failure times are generated conditionally on being greater than the corresponding censoring times, following Royston's principle 20 explained in his Section 5.2. Dorey *et al.* 21 discuss ways for imputing interval censored data, ensuring that the imputed failure times lie in the appropriate intervals. Once the imputed datasets have been created, the parameters of interest are estimated by fitting an analysis model to each of the imputed datasets. Finally, the resulting parameter estimates are combined using Rubin's rules 14, Chapter 9] in the usual way.

The rest of the paper is set out as follows. In Section 2, we describe our motivating example and present the results from a Cox proportional hazards model where the independent censoring assumption is especially suspect. In Section 3, we describe our proposal for relaxing the independent censoring assumption. In Section 4, we apply our methods to our example, in Section 5 we perform a simulation study, and we conclude with a discussion in Section 6.

## 2 Motivating example

Our motivating example is an analysis of a dataset from Watson-Jones *et al*. 22. A total of 821 female workers at recreational facilities in northwestern Tanzania participated in an HIV-prevention trial of herpes simplex virus type 2 (HSV-2) suppressive therapy, where failure is HIV infection. Women were randomised to acyclovir (400 mg twice daily) or placebo and were followed up for up to 12 (*n* = 203) or 30 (*n* = 618) months, depending on phase of enrolment. To be eligible for enrollment, women had to be HSV-2 seropositive, 16–35 years of age, not pregnant or planning a pregnancy in the next 2 years, and not breastfeeding. A total of 679 participants completed follow-up for the primary modified intention-to-treat analysis. In this analysis, the women who become pregnant during the trial are censored at the date of the first positive pregnancy test 22. A summary of the participants' outcomes is shown in the top part of Table 1. The analysis presented by Watson-Jones *et al*. reported no evidence that acyclovir HSV suppressive therapy decreases the incidence of infection with HIV. Because this analysis censors women at the times of pregnancy, the estimands relate to the time to HIV infection in women who are not pregnant.

**Table 1:** Summary statistics for the HIV infection data; there are 821 women in the sample.

Variable	Summary
Lost to follow-up (censored)	142 (17%)
Pregnant (censored)	165 (20%)
Completed follow-up without HIV infection	459 (56%)
or pregnancy (censored)
HIV infection (event)	55 (7%)
Age, mean (SD)	27.4 (5.1)
Drinks per week=0	411 (50%)
Drinks per week=1–9	272 (33%)
Drinks per week=10–29	110 (13%)
Drinks per week=30+	28 (3%)
Lived at site <2years	129 (16%)

It is of interest to know whether any of the baseline variables collected are good predictors of time to infection with HIV. A complication described by Watson-Jones *et al*. is that 165 women became pregnant during the course of the trial. These participants stopped taking the study tablets and were referred to the nearest antenatal clinic. We follow the convention of the primary modified intention-to-treat analysis, where women are censored at the first positive pregnancy test, and so we investigate the risk factors associated with HIV infection among nonpregnant women. An exploratory analysis was performed using Cox proportional hazards models, where the outcome measure was time to infection (in years). Three baseline variables appeared to be good predictors of the hazard of HIV infection: age (at screening), the number of alcoholic drinks per week (for which a categorical variable was used, to avoid assuming a linear effect), and a binary variable indicating that the participant had lived at the screening site for less than 2years. A summary of these covariates is shown in the bottom part of Table 1, and the parameter estimates (log hazard ratios) from the Cox proportional hazards model, obtained by maximising the partial likelihood in the usual way, are shown in Table 2. This analysis suggests that younger women are at greater risk and that consuming alcoholic drinks and participant mobility are also associated with an increased risk of HIV infection. The estimate of *β*_4_ is very similar to, and is slightly smaller than, the estimate of *β*_3_. This, combined with the observation that only 3% of the women are considered heavy drinkers (Table 1), may encourage the collapsing of the last two categories of the drinking-related covariate into a single group. However, the observation that 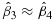 could be dependent on the independent censoring assumption. We will examine whether or not this is the case later in the paper when we relax this assumption.

**Table 2:** Parameter estimates (log hazard ratios) from a proportional hazards model fit to the HIV infection data assuming independent censoring.

Parameter	Baseline covariate	Estimate	Standard error
*β*_1_	Age	−0.084	0.030
*β*_2_	Drinks per week=1–9	0.684	0.342
*β*_3_	Drinks per week=10–29	1.261	0.362
*β*_4_	Drinks per week=30+	1.118	0.568
*β*_5_	Lived at site <2years	0.687	0.301

The results in Table 2 assume independent censoring. This is not very plausible for several reasons. First of all, one of the reasons for censoring is pregnancy. It is quite plausible that pregnancy, and hence censoring, is associated with an increased risk of HIV infection because they both share the common underlying cause of unprotected sexual intercourse. Furthermore, censoring may instead have occurred because the participant has moved away. If greater mobility during the course of the trial is associated with an increased risk of HIV infection, as the fitted proportional hazards model suggests, and the association between moving away and HIV risk is not fully explained by the covariates in Table 2, then this too could invalidate the independent censoring assumption. However, the inclusion of alcohol-related covariates in the model might be thought to make the independent censoring assumption more plausible because, if women with high alcohol intake have more chaotic lifestyles, this high intake of alcohol could be thought to be associated with both censoring and HIV infection. The most obvious concern, however, is that censoring may be associated with a permanent change, and in particular an increase, in the risk of HIV infection at around the time it occurs, which would invalidate the independent censoring assumption. In the next section, we develop our model, and an accompanying procedure for making inferences, so that the independent censoring assumption made by the analysis reported in Table 2 may be relaxed.

## 3 Relaxing the independent censoring assumption

In this section, we develop our model that relaxes the independent censoring assumption. We begin by describing our full model that makes no assumptions about the conditional distribution of the failure time given that it is after the censoring time. We then consider a simplification of this model so that it assumes a step change at the time of censoring, because this simple model is both amenable to sensitivity analysis and reflects our intuition, in the context of our example that there is a change in the hazard of failure when censoring occurs.

### 3.1 Notation

We assume that both the time to failure, *T*_*i*_, and time to censoring, *C*_*i*_, for the *i*th participant are continuous random variables. We treat failure and censoring as competing risks, so we observe *Y*_*i*_= min(*T*_*i*_,*C*_*i*_) and *δ*_*i*_, where *δ*_*i*_=1 if *T*_*i*_<*C*_*i*_ and *δ*_*i*_=0 otherwise. We also assume that all participants provide complete data on the regression covariates and the variables on which we stratify, which we denote as vectors *Z*_*i*_ and *S*_*i*_, respectively. For our motivating example in the previous section, *Z*_*i*_ consists of the baseline covariates shown in Table 2, and we do not stratify, so *S*_*i*_ is empty. We denote any additional variables that describe the hazard of failure after censoring as *W*_*i*_. We use *h*(*t*) to denote the hazard of failure and *H*(*t*) to denote the cumulative hazard of failure. We describe the association between *T*_*i*_ and *C*_*i*_ and so allow departures from independent censoring, by modelling the conditional distribution of *T*_*i*_ given *C*_*i*_.

### 3.2 The independent censoring assumption

We follow Fleming and Harrington, who interpret independent censoring as meaning that the hazard of failure at time *t* is equal to the hazard of failure at time *t* given that censoring has not yet occurred 4, pp. 26–27]. In our notation, independent censoring is satisfied if 

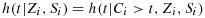
1
for all participants, where *h*(*t*|*C*_*i*_>*t*,*Z*_*i*_,*S*_*i*_) is the conditional hazard of failure *given that censoring has not yet occurred*. Condition (1) involves covariates, and so this assumption may be more, or less, plausible depending on the covariates included in the model. The independent censoring assumption implies that maximising the partial likelihood in the usual way provides valid inference for both *h*(*t*|*Z*_*i*_,*S*_*i*_) and *h*(*t*|*C*_*i*_>*t*,*Z*_*i*_,*S*_*i*_) when fitting Cox models 4, pp. 139–140], where the first of these hazard functions is usually of scientific interest. The independent censoring assumption in (1) is slightly weaker than the assumption of statistical independence of *T*_*i*_ and *C*_*i*_ 4, p. 27], although the latter is often stated as justifying the use of standard methods. Hence, the commonly stated assumption that *T*_*i*_ and *C*_*i*_ are independent is a sufficient, but not necessary, condition for justifying standard methods.

### 3.3 Relaxing the independent censoring assumption

Our aim is to relax the independent censoring assumption for the Cox proportional hazards model, and we begin by assuming that the conditional hazard of failure, given that censoring has not yet occurred, is given by 

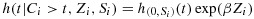
2
where 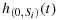 is the baseline hazard function, stratified by variables *S*_*i*_, and *β* is the row vector of regression coefficients. Model (2) can be fitted to the observed data *Y*_*i*_, *δ*_*i*_, *Z*_*i*_ and *S*_*i*_, *i* = 1,=,*n*, using the partial likelihood in the conventional way. Model (2) therefore provides an interpretation of the hazard ratios in fitted Cox models where the independent censoring assumption need not be true, and so it provides a vital link between our methodology and applied work where Cox proportional hazards models have been fitted despite doubt about the independent censoring assumption. If a fully parametric model were instead used for (2), then this model could be fitted using the full likelihood.

In order to extend model (2) to model the conditional hazard given *C*_*i*_<*t*, *C*_*i*_ and the covariates, we assume 

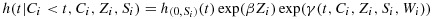
3
where *γ*(*t*,*C*_*i*_,*Z*_*i*_,*S*_*i*_,*W*_*i*_) is the log hazard ratio at time *t* of censored and uncensored individuals with equal *C*_*i*_*Z*_*i*_, *S*_*i*_ and *W*_*i*_. We allow *γ*(·) to be a function of additional variables *W*_*i*_, to indicate that this function could also depend on any other variables. For example, *W*_*i*_ could include the reason for censoring. Because we do not observe failure times after censoring, the data provide no information about *γ*(*t*,*C*_*i*_,*Z*_*i*_,*S*_*i*_,*W*_*i*_), so the analyst must make an untestable assumption about what this function is in order to apply model (3).

If *γ*(*t*,*C*_*i*_,*Z*_*i*_,*S*_*i*_,*W*_*i*_) = 0 for all *i*, then *h*(*t*|*C*_*i*_<*t*,*C*_*i*_,*Z*_*i*_,*S*_*i*_) = *h*(*t*|*C*_*i*_<*t*,*Z*_*i*_,*S*_*i*_) = *h*(*t*|*C*_*i*_>*t*,*Z*_*i*_,*S*_*i*_), and so the independent censoring assumption (1) is true. However, if *γ*(*t*,*C*_*i*_,*Z*_*i*_,*S*_*i*_,*W*_*i*_) ≠ 0 for any *i*, then the independent censoring assumption is false. Hence, in our conceptual framework, any departure from *γ*(*t*,*C*_*i*_,*Z*_*i*_,*S*_*i*_,*W*_*i*_) = 0 provides an alternative to the assumption of independent censoring.

### 3.4 A simplified model

To make convenient use of model (3) in a sensitivity analysis, we will consider the simplified form *γ*(*t*,*C*_*i*_,*Z*_*i*_,*S*_*i*_,*W*_*i*_) = *γ*_*i*_, that is, *γ*(·) does not depend on time. This means that participants' hazard functions receive a step change at the time of censoring. The parameters *γ*_*i*_ represent log hazard ratios associated with censoring in the model for the time to failure, where censoring is interpreted as a time-dependent binary covariate. If *γ*_*i*_>0, then the *i*th participant is at an elevated risk of failure after censoring, but if *γ*_*i*_<0, then this participant is instead at a reduced risk after censoring. Hence, both harmful and protective effects of censoring are possible. This simplified model has some similarities to Letué's shock model 23. More general, time-dependent, forms of *γ*(*t*,*C*_*i*_,*Z*_*i*_,*S*_*i*_,*W*_*i*_) are feasible, and we return to this possibility in the discussion. We perform a sensitivity analyses using the *γ*_*i*_ as sensitivity parameters.

### 3.5 Creating imputed datasets using proportional hazards models

We use multiple imputation to create datasets where there are no censored observations during the follow-up period. When using multiple imputation, we must take the uncertainty in the imputation model into account, and here, this includes both the regression parameters *β* and the baseline hazard function. Bootstrapping 16 is a convenient way to take into account the uncertainty in the form of a semi-parametric imputation model such as (2) and may be used in conjunction with multiple imputation 24. Here, we sample subjects with replacement to create *m* bootstrap samples, one for each subsequent imputed dataset, where we include both censored and uncensored participants in this sampling. In our application, we sampled subjects with replacement from the entire sample when creating our bootstrap samples, but in situations where the model involves different treatment groups, or other structural strata, we would usually sample with replacement within these strata.

We estimate the regression parameters and cumulative baseline hazard function from the bootstrap samples, using the Breslow estimator for the latter. The resulting estimates, 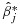 and 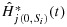, *j* = 1,=,*m*, are used when imputing failure times for censored subjects in the *m* imputed datasets. As we will see later, Cox regression models are then fitted to the imputed datasets. By imputing censored data under specific, user-defined, departures from the censoring at random assumption, we are able to avoid the large biases that can result from incorrectly making this assumption.

We impute the participants' censored failure times conditional on their observed data, which comprises their covariates, *C*_*i*_ and *δ*_*i*_=0, as follows. The hazard given *S*_*i*_, *Z*_*i*_, *C*_*i*_ and *δ*_*i*_=0 is zero for *t* < *C*_*i*_ and is given by (3) for *t* > *C*_*i*_, and so the hazard from which we draw imputed failure times for the *i*th participant in the *j*th imputed dataset is 

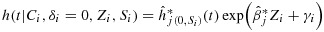

if *t* > *C*_*i*_ and *h*(*t*|*C*_*i*_,*δ*_*i*_=0,*Z*_*i*_,*S*_*i*_) = 0 if *t* < *C*_*i*_. A convenient way to impute a failure time from this distribution is to simulate a time *A*_*ij*_, from the time of censoring to the failure time, from the distribution with hazard

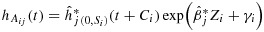
5
and then calculate the imputed failure times for censored participants in the *j*th imputed dataset as *T*_*ij*_=*C*_*i*_+*A*_*ij*_. To use the method proposed by Bender *et al.* 25 to simulate times from (5), we require the corresponding cumulative baseline hazard function. The cumulative baseline hazard function of *A*_*ij*_ is 

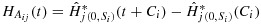

We therefore simulate *A*_*ij*_ from a Cox model with cumulative baseline function 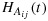 and linear predictor 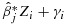. Following Bender *et al.* 25, we generate *U*_*ij*_∼Unif(0,1) and then calculate 

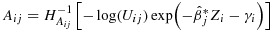
7
However, this requires inverting 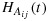, which is not immediate because the cumulative hazard function in (7) is a step function. We define the inverse 

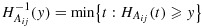

so that the failure time *T*_*ij*_=*C*_*i*_+*A*_*ij*_ can be imputed for censored participants. If 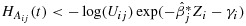 for all *t*, then we impute a censored failure time at the end of the follow-up period. Because 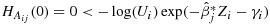, we never simulate *A*_*ij*_=0 and hence deaths immediately after censoring, when using any finite value of *γ*_*i*_. This is appropriate because we know that failures occur after the censoring times.

If instead *γ*(*t*,*C*_*i*_,*Z*_*i*_,*S*_*i*_,*W*_*i*_) is a function of *t*, then the failure times would be simulated from a distribution with a hazard function of the form in (3) for *t* > *C*_*i*_, and *h*(*t*|*C*_*i*_,*δ*_*i*_=0,*Z*_*i*_,*S*_*i*_) = 0 for *t* < *C*_*i*_. This would require an alternative method for imputing failure times. We return to this issue in the discussion.

If a parametric proportional hazards model were used instead of a Cox model in (2) and (3), then we would create multiple imputed datasets in a very similar way, except that to save computation time and remove the need to bootstrap, we could simulate the 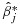 and the parameters of the baseline hazard function from the estimated asymptotic normal distribution of the maximum likelihood estimator, as in more conventional multiple imputation procedures. Our procedure for creating imputed datasets is therefore simplified when using a parametric proportional hazards model instead of a Cox model in (2) and (3). We could also use this type of more conventional multiple imputation procedure in conjunction with the Cox model if we were prepared to ignore the uncertainty in the estimated baseline hazard function. However, it is very difficult to see how the uncertainty in this function could be taken into account when using an alternative to our proposed bootstrap procedure. An advantage of ignoring the uncertainty in the estimated baseline hazard function, and using more conventional multiple imputation procedures, would be to greatly reduce the computational demand, and so this option might be deemed preferable in very large datasets.

### 3.6 The analysis of the imputed datasets

Having created *m* imputed datasets, the standard multiple imputation procedure is used to obtain parameter estimates: each imputed dataset is analysed separately using a standard method for survival analysis, and the resulting estimates are combined using Rubin's rules 14, Chapter 9.2].

We propose fitting the standard Cox proportional hazards model 

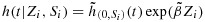
9
to the imputed datasets, that is, (9) is the analysis model in the multiple imputation procedure. Although the unconditional (on *C*_*i*_) model (9) is a Cox proportional hazards model of the same form as the conditional (on *C*_*i*_>*t*) model (2), it has different parameter values unless all *γ*_*i*_=0. We follow the usual procedure and fit the analysis model (9) to each of the imputed datasets and combine the resulting estimates of *β* using Rubin's rules to provide estimates that neither assume independent censoring nor require that the estimands are interpreted conditionally on censoring not having occurred.

The assumption that the model in Section 3.3 and the analysis model (9) adequately describe the conditional and unconditional distributions of the failure times, respectively, must be carefully checked in practice. Letué's 23 formulae for her marginal survivor functions in her Remark 2.1, interpreting her two events as failure and censoring, show that, in general, models (2) and (9) cannot both be true. Although this incompatibility is a theoretical concern, in practice, we are content to apply statistical models when they describe the data reasonably well. Diagnostics to assess model fit are a crucial component of all applied statistical work, but this is especially important here where we are using models that we know are incompatible. We suggest using residuals to assess whether (2) and (9) are adequate, where the residuals used for assessing (2) are from a Cox model fit to the observed data and those for assessing (9) are from Cox model fits to the imputed datasets. We use the Schoenfeld residuals 26, but other types of residuals could also be used for this purpose.

If a parametric proportional hazards model was used instead of a Cox model in (2) and (3), then this same type of parametric model could also be used as the analysis model in (9), provided that this parametric model adequately describes both the original dataset and the imputed dataset. In situations where a parametric model were used in (2) to analyse the observed data, but this type of model provides a poor fit to the imputed datasets, a Cox proportional hazards model could be used as the analysis model in order to better describe the imputed data. If the Cox model does not adequately describe the imputed datasets, then other analysis models should be considered, and we return to this issue in the discussion.

## 4 Application to the HIV-prevention trial

We now show how to apply our method in practice by extending the analysis in Table 2. The association between censoring and HIV infection, which need not be causal, could be due to the common cause of unprotected sexual intercourse for both pregnancy (which results in censoring) and HIV infection. Alternatively, this association could be because of greater participant mobility at the time of censoring, a change in behaviour after censoring, or a combination of these or other reasons. The step changes assumed in our model may be more, or less, plausible in other contexts, and we return to this issue in the discussion.

We interpret the estimates from our procedure as estimating effects where censoring before the end of the trial cannot occur. Hence, our estimates quantify the risk factors associated with HIV infection among nonpregnant women, as the trial originally intended.

### 4.1 A sensitivity analysis

**Figure 1:**
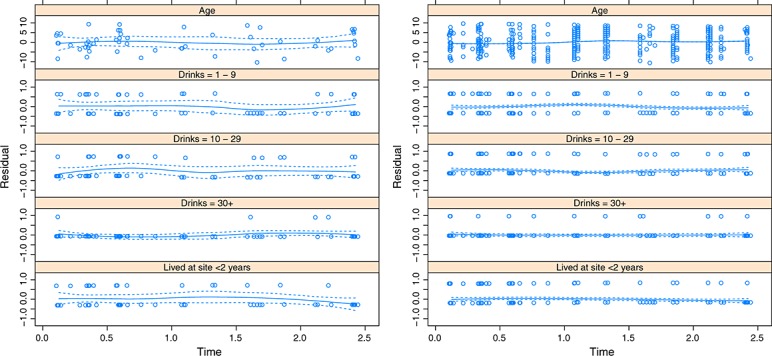
Left: Schoenfeld residuals for the five parameters in Table 2, from fitting model (2) to the incidence of HIV data. Right: Schoenfeld residuals for the five parameters in Table 2, from fitting model (9) to the first imputed dataset using *γ* = 5. LOWESS smoothers are also shown, together with ± 2 standard deviation confidence bands.

First of all, we assess whether model (2) is adequate for our data. The left panel of Figure 1, which shows plots of the Schoenfeld residuals from the fitted model shown in Table 2, suggests that model (2) is adequate for this purpose because the residuals appear to be centred at zero and there is no evidence of trends over time. Of course, diagnostics such as these should routinely accompany all regression models, and under the assumption of independent censoring, Figure 1 would reassure the analyst that model (2) is suitable for the unconditional failure times.

In order to relax the independent censoring assumption and perform an illustrative sensitivity analysis, we take *γ*_*i*_=*γ* for all *i*, and we allow *γ* to take the values −3,−2,−1,=,10; this includes a wide range of possibilities as shown later. This illustrative analysis assumes that censoring for any reason is associated with the same step change in the hazard of failure for all participants. We relax this assumption in Section 4.2. We could plausibly use a shorter range of values of *γ* in a sensitivity analysis, but here, we intend to demonstrate that large positive and negative *γ* effectively impute ‘censoring=immediate infection’ and ‘censoring=never infected’, respectively. The most obvious concern is that there may be a positive association between HIV infection and censoring. Hence, the suspicion is that *γ* > 0, but some negative values are also investigated in order to illustrate the methodology and perform a very thorough sensitivity analysis. The parameter *γ* denotes the log hazard ratio comparing censored with uncensored participants. By comparing this parameter to the estimates in Table 2, the magnitude of the effect of censoring can be compared with the covariate effects of interest.

We generated a relatively large number *m* = 200 of imputed datasets for each value of *γ* in order to reduce the Monte Carlo error, but smaller values of *m* are usually considered acceptable. The intended maximum follow-up period was 30months, and the greatest observed time in the dataset was a censored time of around 33months. We took the end of the follow-up period to be 3years so that imputed failure times were taken to be censored at 3years if 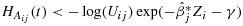 for all *t*, as explained in Section 3.5.

Before combining the results using Rubin's rules, however, we must check that model (9) adequately describes the failure times in the imputed datasets. This involves examining a selection of, and ideally all, the residual plots for the analyses of the imputed datasets. A representative set of residual plots are shown in the right-hand panel of Figure 1, which shows the residuals for the first imputed dataset using *γ* = 5. The confidence bands are tighter in the right-hand panel of Figure 1, because *γ* = 5 imputes many infections, and so, there are considerably more residuals to estimate the smoothed fit in the right-hand panel. Whilst recognising that it is possible to improve upon the model fit by considering more complex models, our examination of residual plots such as those shown in Figure 1 reassure us that model (9) provides a reasonable description of the failure times in the imputed datasets.

**Figure 2:**
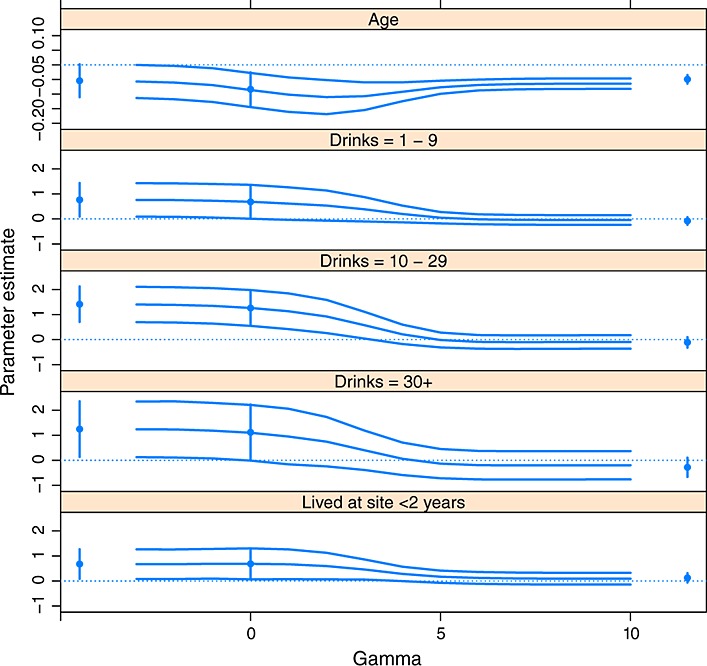
Results from the sensitivity analysis for the HIV incidence data. The curves show the point estimates and 95% confidence intervals for the log hazard ratios associated with the covariates indicated. Inferences from the standard analysis assuming independent censoring from Table 2 are shown at *γ* = 0. Inferences are also shown from imputing all censored observations as ‘never infected’, and all censored observations as ‘immediate failures’, at the left-hand and right-hand sides of the plots, respectively.

We are therefore prepared to use Rubin's rules to combine the estimates applying model (9) to the imputed datasets, and the results are shown in Figure 2 for all five regression parameters shown in Table 2. Note that a different vertical axis is used for the first regression coefficient in Figure 2. The curves connect estimates for *γ* =− 3,−2,−1,=,10, and 95% confidence intervals, obtained as the estimate plus and minus 1.96 standard errors, are also shown. The same 200 draws of 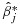 and 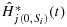 were used across all 14 values of *γ*.

The results from three further analyses are shown in Figure 2, where the estimates are displayed as solid points and the 95% intervals are vertical lines. At the left-hand side of the plots in Figure 2, we show the results from the analysis where all censored participants are treated as ‘never infected’; this results in a single ‘imputation’ procedure where all censored participants' censoring times are set equal to the maximum time of 3years. At *γ* = 0, we show the results from the usual analysis assuming independent censoring, as also shown in Table 2. At the right of the plots, we show the results assuming participants who are censored are immediately infected with HIV; this is another single ‘imputation’ procedure where all participants' censoring indicators *δ* are set equal to one and the resulting data are analysed. Hence, the results at the left-hand and right-hand sides of the plots show the results from two very extreme departures from independent censoring. Our sensitivity analysis enables us to consider all ‘shades of grey’ between these two extremities.

The results in Table 2 and using our procedure with *γ* = 0 are in very good agreement (Figure 2), as anticipated because these analyses make the same assumptions. This shows that our method provides appropriate inferences under the independent censoring assumption and validates our approach. However, the proposed approach uses multiple imputation and is subject to Monte Carlo error, so non-identical numerical results are inevitable. Recalling that *γ* is the log hazard ratio of censored and uncensored individuals, as *γ*→−*∞*, the results tend towards ‘censored=never infected’. Figure 2 shows that *γ* =− 3 is sufficient to produce results that are close to this limiting result, because the HIV infection rate is not very high (Table 1). The results for *γ* = 10 are in reasonable agreement with the analysis that assumes ‘censoring=immediate infection’ but are not in perfect agreement because the proposed procedure never imputes immediate failures after censoring for finite *γ* as explained in Section 3.5. However, increasing *γ* further did not change the estimates much, and an analysis assuming ‘censoring=immediate infection’ can easily be performed. These observations mean that the values *γ* =− 3,−2,−1,=,10 used in the sensitivity analysis include a very wide range of possibilities that allow us to explore how rapidly the extreme scenarios are approached. Figure 2 shows that as *γ* increases, the lengths of the confidence intervals shorten, which can be explained by the additional information obtained by assuming that censoring is strongly associated with failure so that more failures are simulated in the imputed datasets.

The inference that age is an important predictor in the analysis assuming independent censoring, presented in Table 2, is reinforced by the sensitivity analysis because a large and statistically significant effect over the entire range of possibilities is seen. The inferences that the consumption of alcoholic drinks is associated with higher infection rates is more sensitive however; as *γ* increases, the magnitude of the corresponding three estimates, and their statistical significance, falls. Recalling that the suspicion is that *γ* > 0, the sensitivity analysis greatly reduces the strength of evidence that consuming alcohol is an important predictor. The inference that mobility is associated with an increase in the infection rate is insensitive to small departures from independent censoring, but for large *γ*, both the magnitude and the statistical significance of this effect drop sharply. A local sensitivity analysis would conclude that inferences for this regression parameter are not sensitive to relaxing the independent censoring assumption, but our global sensitivity analysis shows that these inferences are sensitive to larger departures from independent censoring. The observation that 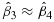, discussed in (3)Section 2, still holds in this and the other sensitivity analyses we performed. This strengthens the case for collapsing the drinking-related covariate into three groups.

### 4.2 A sensitivity analysis assuming independent censoring unless censored due to pregnancy

**Figure 3:**
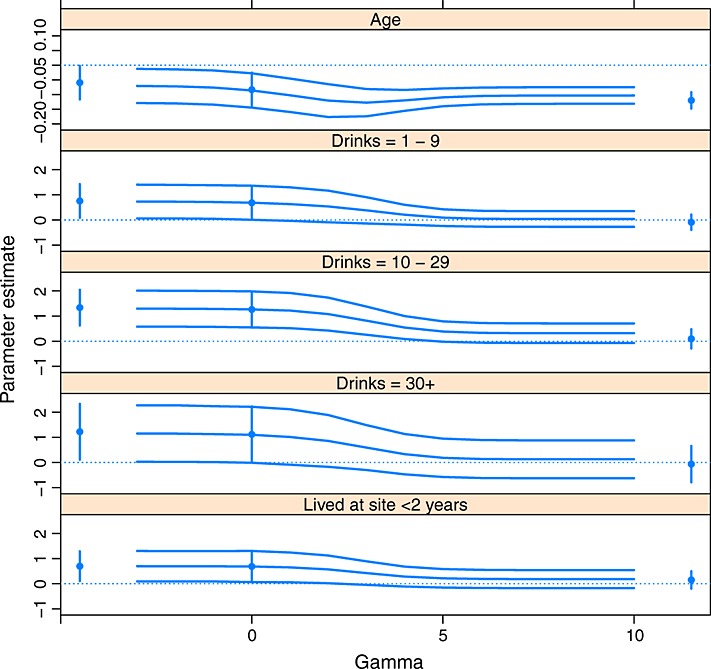
Results from the sensitivity analysis for the HIV incidence data assuming independent censoring *unless censored due to pregnancy*. The curves show the point estimates and 95% confidence intervals for the log hazard ratios associated with the covariates indicated. Inferences from the standard analysis assuming independent censoring from Table 2 are shown at *γ* = 0. Inferences are also shown from imputing all censored (*due to pregnancy*) observations as ‘never infected’, and all censored (*due to pregnancy*) observations as ‘immediate failures’, at the left-hand and right-hand sides of the plots, respectively.

The assumption that *γ*_*i*_=*γ* for all *i* is not very plausible because participants are censored for different reasons. In particular, for participants who are administratively censored because the end of their follow-up period is reached, one can safely assume *γ*_*i*_=0. In our example, we have two main types of non-administrative censoring: censoring due to pregnancy and censoring for other reasons. Censoring due to pregnancy might be thought to be more positively associated with the change in rate of HIV infection than other reasons for censoring. Hence, in Figure 3, we show the results from a sensitivity analysis where *γ*_*i*_=*γ* for participants censored due to pregnancy and *γ*_*i*_=0 for all other participants. Alternative values of *γ*_*i*_ for participants non-administratively censored for reasons other than pregnancy could also be used however, perhaps to reflect the fact that their censoring may be associated with increased mobility and therefore a higher infection rate. Alternatively, participants could become pregnant after censoring, and an alternative value of *γ*_*i*_ could be used to reflect, but not directly model, that possibility. Further possibilities include allowing *γ*_*i*_ to depend on further covariates and participants' censoring times. In the supporting information, we provide an illustrative sensitivity analysis, where *γ*_*i*_ is a function of the mobility baseline covariate and where the sign of *γ*_*i*_ is allowed to be positive for some participants and negative for others. Further analyses could also be performed, for example, by using different *γ*_*i*_ for all three types of censoring in Table 1. Hence, a very wide range of possibilities could be explored. Our data are not freely available, but the R code used to implement the sensitivity analysis performed in (12)Section 4.1 is available from the first author on request.

The inferences assuming independent censoring are less sensitive to departures from this assumption now that the independent censoring assumption is assumed to be true for many participants. The confidence intervals for large positive *γ* are wider in Figure 3 than in Figure 2 because fewer failures are imputed. Model (9) was deemed suitable for the analysis of the imputed datasets from an examination of the Schoenfeld residuals in the way described in Section 4.1.

## 5 Simulation study

A simulation study, loosely based on the HIV trial data, was used to investigate the performance of the proposed method. Every simulated dataset involved 1000 participants, and there was a single categorical covariate *Z*. We generated *Z*_*i*_=0 with probability 0.5, *Z*_*i*_=1 with probability 0.3, and *Z*_*i*_=2 with probability 0.2; this covariate was intended to mimic the ‘Drinks per week’ categorical covariate in the HIV trial, with the last two categories collapsed into a single group. To simulate outcomes, we generated *C*_*i*_∼Exp(0.3) and *T*_*i*_∼Exp(*λ*_*i*_) independently, where Exp(*λ*) denotes an exponential distribution with rate *λ*; we used *λ*_*i*_=0.03 if *Z*_*i*_=0, *λ*_*i*_=0.05 if *Z*_*i*_=1, and *λ*_*i*_=0.09 if *Z*_*i*_=2. These rates produce censoring and failure rates that are similar to those found in the HIV data. We set *Y*_*i*_= min(*T*_*i*_,*C*_*i*_,3) and *δ*_*i*_=1 if *T*_*i*_<*C*_*i*_ and *T*_*i*_<3, and otherwise, we set *δ*_*i*_=0. This means that the follow-up period in the simulation study was 3years, again imitating the HIV trial. The simulation study was performed under the assumption that our imputation model (3) is correct and that *γ*(·) = *γ* for all *i*. A thousand simulated datasets were produced for each of *γ* =− 2,−1,0,1,2,3,4,5. From the sensitivity analysis of the HIV data in Section 4.1, these values of *γ* can be seen to cover quite a wide range of possibilities.

To calculate the *true* log hazard ratios associated with the covariate *Z* when *γ* ≠ 0 (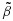 in Equation  (9), we simulated for each value of *γ* a single dataset with a million participants. We augmented these simulated datasets with failure times for all participants as 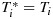 if *T*_*i*_<*C*_*i*_ and 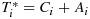 if *T*_*i*_>*C*_*i*_, where *A*_*i*_∼Exp(*λ*_*i*_ exp(*γ*)). We then fitted a Cox model using the 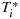 as failure times, after first censoring 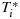 at 3years, and took the regression coefficients (two log hazard ratios comparing *Z* = 1 and *Z* = 2 to the reference category *Z* = 0) as the true log hazard ratios. This data generating process follows model (3) over the follow-up period with *γ*(*t*,*C*_*i*_,*Z*_*i*_,*S*_*i*_,*W*_*i*_) = *γ*. For *γ* = 0, the true log hazard ratios are log(0.05/0.03) and log(0.09/0.03).

In order to assess the extent of the bias resulting from incorrectly assuming independent censoring, we estimated the two log hazard ratios comparing *Z*_*i*_=1 and *Z*_*i*_=2 to *Z*_*i*_=0, using the simulated outcome data *Y*_*i*_ and *δ*_*i*_ and a standard Cox regression. In order to assess the performance of the proposed method, we also estimated these two hazard ratios using the proposed method *and the correct value of γ*.

To estimate biases, we took the difference between the average estimated log hazard ratios and the true log hazard ratios. Furthermore, the proportion of the 1000 95% confidence intervals that contain the true log hazard ratios provides an estimate of the coverage probabilities produced using the proposed method and standard Cox regression.

To reduce the computational burden, we used *m* = 10 imputed datasets when implementing our proposed method; *m* = 200 was used in the application in Section 4, but Rubin's rules give valid inference with smaller *m*, such as *m* = 10, so this is perfectly acceptable value to establish the statistical properties of the method in a simulation study. However, small *m* will be subject to greater Monte Carlo error than large *m*; hence, for applications (as here), we may well prefer to use a larger value of *m*.

The results are shown in Table 3. The Monte Carlo standard errors of the biases for each value of *γ* were obtained from the empirical standard deviations of the estimated log hazard ratios using the two competing approaches and the reported standard errors from the Cox regressions fitted to the very large datasets that were used to calculate true values. The Monte Carlo standard errors for the proposed method become smaller for large *γ* because the imputed datasets contain more failures as *γ* increases. The Monte Carlo standard errors at *γ* = 0 are slightly smaller than those for other values of *γ* because there is no uncertainty in the true values of the regression parameters when the independent censoring assumption is true.

**Table 3:** Results from the simulation study.

	First parameter:		First parameter:		Second parameter:		Second parameter:	
	bias (MCSE)		coverage		bias (MCSE)		coverage	
*γ*	MI	IC	MI	IC	MI	IC	MI	IC
−2	0.007 (0.012)	0.012 (0.012)	0.960	0.958	0.011 (0.011)	0.020 (0.011)	0.948	0.952
−1	0.003 (0.012)	0.007 (0.012)	0.947	0.948	0.006 (0.011)	0.011 (0.011)	0.955	0.950
0	0.018 (0.009)	0.019 (0.009)	0.953	0.959	−0.001 (0.008)	0.000 (0.008)	0.960	0.961
1	0.017 (0.011)	0.025 (0.010)	0.937	0.950	0.015 (0.010)	0.032 (0.010)	0.941	0.939
2	−0.013 (0.009)	0.036 (0.010)	0.930	0.953	−0.014 (0.009)	0.120 (0.009)	0.931	0.936
3	−0.008 (0.008)	0.152 (0.009)	0.920	0.915	−0.003 (0.007)	0.397 (0.009)	0.926	0.655
4	−0.006 (0.005)	0.301 (0.009)	0.928	0.800	0.008 (0.006)	0.704 (0.009)	0.930	0.222
5	0.004 (0.004)	0.409 (0.009)	0.941	0.665	0.012 (0.005)	0.861 (0.009)	0.935	0.096

‘MI’ indicates that the proposed multiple imputation procedure (using the correct value of *γ*) is used, and ‘IC’ indicates that a standard Cox proportional hazards model assuming independent censoring is used. ‘First parameter’ indicates that the results are for the log hazard ratio of *Z* = 1 relative to *Z* = 0; ‘second parameter’ indicates that the results are for the log hazard ratio of *Z* = 2 relative to *Z* = 0. Monte Carlo standard errors (MCSE) of the estimated biases are provided in parentheses, and ‘coverage’ denotes the estimated coverage probabilities of 95% confidence intervals. A total of 1000 simulated datasets were produced for each value of *γ*, and ‘MI’ and ‘IC’ were applied to the same sets of simulated datasets.

The results in Table 3 show that the proposed method performs well across the entire range of *γ*: there is no evidence of bias in the estimates, and the actual coverage probability of all 95% confidence intervals are reasonably close to the nominal levels. By comparison, the estimates from standard Cox regression models that assume independent censoring become extremely biased for large *γ*, and the coverage probabilities of 95% confidence intervals suffer very badly because of this. This is because larger values of *γ* result in true hazard ratios are much closer to zero and hence positive bias from standard Cox regressions fitted to observed data.

## 6 Discussion

We have proposed and implemented a method for assessing the sensitivity of the inferences made from Cox proportional hazards models to the independent censoring assumption. We have achieved this by using a relatively simple model, but one that reflects the concern that censoring is associated with a sudden change in the risk of failure. This type of assumption is perhaps most plausible in the context of trials where censoring is due to participants ceasing to take their randomised treatment, but our approach provides a relatively straightforward and direct way to assess the sensitivity of inferences to the independent censoring assumption wherever Cox models are used. For example, a sharp, but continuous, change in the hazard function at around the time of censoring may be thought more plausible than independent censoring, which can be approximated by our method. Our methodology makes the assumptions required conceptually straightforward and accessible to applied researchers.

We have explained how our methodology could be adapted for use when fully parametric proportional hazards models, such as the Weibull model, are used. Perhaps the main difficulty that we can anticipate when using parametric models is that the form of the model may not be suitable as the analysis model for the imputed datasets, despite the fact that it was adequate for the observed data. This is because, by fully specifying the form of the failure distribution, there is greater capacity for parametric models to inadequately describe the imputed datasets. Alternative analysis models should be investigated in situations where the proposed analysis model is found not to describe the imputed data sufficiently well. In particular, if a parametric model is used and provides a satisfactory fit for the observed data, but not for the imputed datasets, Cox models could be used for the analysis of the latter. Pragmatically, there is no requirement that the models used for the observed and imputed datasets are of the same form, but all models used must describe the various datasets well. If a Cox model does not adequately describe the imputed datasets, then alternatives to proportional hazards models could be considered, where we have an abundance of possibilities to choose from 27,28. If however, in extreme cases, no model can be found to describe the failure times in the imputed datasets, then this means that the attempt to describe the distribution of failure times using the proposed model have failed.

More sophisticated models for the association between the failure and censoring mechanisms might be thought more plausible in some contexts, including the HIV trial we use as our example here, and we hope that our ideas will encourage the further development of models of this kind. By using more complicated forms of *γ*(*t*,*C*_*i*_,*Z*_*i*_,*S*_*i*_,*W*_*i*_), more subtle types of departure from independent censoring could be explored. The data provide no information about failure times after censoring, however, so any parametric form for this function makes untestable assumptions. More generally, when *γ*(·) depends on *t*, the failure time *T*_*ij*_ could be imputed as the solution of the equation 

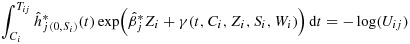

where, as in (7), an exact solution for Cox models is not possible because 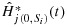 is then a step function and a convention for approximately solving this equation for *T*_*ij*_ is required. The range of values of *γ*_*i*_, and more generally the form of *γ*(*t*,*C*_*i*_,*Z*_*i*_,*S*_*i*_,*W*_*i*_), should ideally be elicited from subject experts, but in situations where this is not possible, a wide range of possibilities can be examined as in Section 4. We could also consider giving the *γ*_*i*_ a distribution and elicit this from subject experts. In addition to making untestable assumptions about the hazard of failure after censoring, our approach also makes testable assumptions about the hazard of failure before censoring. Standard methods should be used to assess these testable assumptions, and we have used residuals for this purpose.

Our methodology is concerned with right censoring, but left and interval censoring are also encountered in practice. Another common extension is the use of time-dependent variables. Both of these additional complications require extensions of our methodology, and we leave these issues as possibilities for future work. However, the proposed approach does handle stratification, so many Cox models fit into our framework. In particular, typical proportional hazards models resulting from randomised controlled trials, where there is typically just a single parameter for the treatment effect, are incorporated. The results from randomised controlled trials are likely to be most sensitive to the *γ*_*i*_ differing across trial arms; if we have similar censoring mechanisms and values of *γ*_*i*_ in both treatment arms, then we can anticipate that inferences from standard analyses will be similar to those using our method.

By conceptualising the censored failure times as missing data, we have essentially turned non-independent censoring into a missing data problem. Because our proposal clearly distinguishes between the parts of the model that are identifiable and those that are not, it is akin to a pattern mixture approach [14, Chapter 3.6]. Because we have used multiple imputation, our approach provides a natural context for also accommodating missing covariates. We leave the best way to do this in practice as an open question, but the methods described by Carpenter and Kenward [29, Chapter 8] provide suitable starting points.

In summary, we believe our proposal provides a practical, flexible approach for exploring the sensitivity of inferences from plausible departures from the ubiquitous assumption of independent censoring with the Cox proportional hazards model.

## Supporting Information

Supporting info item
