# Supplementary material for: Relaxing the independent censoring assumption in the Cox proportional hazards model using multiple imputation
Source: Stat Med. 2014 Jul 25;33(27):4681–94. doi: 10.1002/sim.6274 (PMC4282781; doi:10.1002/sim.6274)
Supplement: Supplementary file 1 — Supporting info item [file sim0033-4681-sd1.pdf]

Research Article

Statistics in Medicine

Received XXXX

(www.interscience.wiley.com) DOI: 10.1002/sim.0000

Supplementary materials for: "Relaxing the independent censoring assumption in the Cox proportional hazards model using multiple imputation"

1. A further sensitivity analysis

A very wide range of possibilities are available because γ_i may be different for every participant. For example, γ_i could be positive for some participants and negative for others, and γ_i could further depend on covariates and/or the censoring time. In figure 1 we show the results where γ_i is as in figure 3 in the main paper, but where participants who have lived at site for two or more years have further had their γ_i reduced by 2. Hence living at site for two or more years has a further protective effect after censoring.

Hence we take $\gamma_i = \gamma$ for participants who are censored due to pregnancy and have lived at site for less than two years, $\gamma_i = \gamma - 2$ for participants who are censored due to pregnancy and have lived at site for two or more years, $\gamma_i = 0$ for participants who are censored for reasons other pregnancy and have lived at site for less than two years and $\gamma_i = -2$ for participants who are censored for reasons other pregnancy and have lived at site for two or more years. This means that different participants may have positive or negative γ_i and that $\gamma(\cdot)$ is a function of one the covariates included in the Cox regression.

The resulting sensitivity analysis is shown in figure 1. Now living at site for two or more years is associated with a further protective effect after censoring has occurred. This sensitivity analysis suggests that the inferences for particular parameters in the Cox regression can be especially sensitive when $\gamma(\cdot)$ is a function of the corresponding covariate. Hence we encourage exploring this possibility in situations where it is plausible that $\gamma(\cdot)$ is a function of one or more of the covariates.

Copyright © 2009 John Wiley & Sons, Ltd.

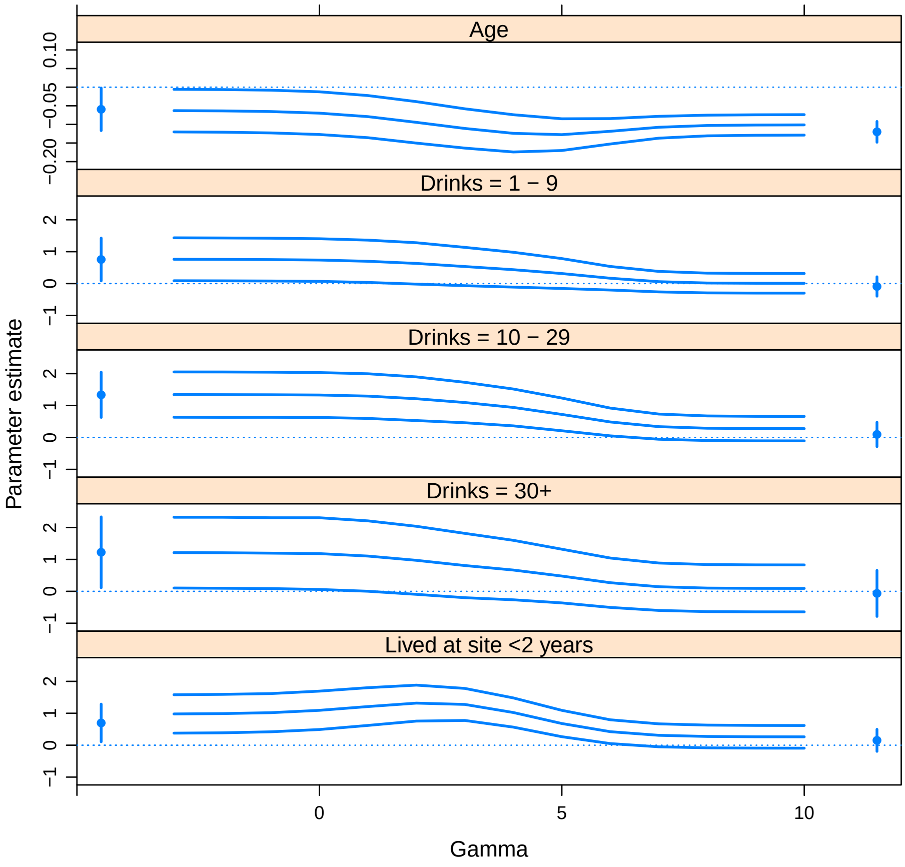

Figure 1. Results from the sensitivity analysis for the HIV incidence data, where γ_i is as in figure 3 of the main paper, but participants who have lived at site for 2 or more years have further had their γ_i reduced by 2. The curves show the point estimates and 95% confidence intervals for the log hazard ratios associated the covariates indicated. Inferences are also shown from imputing all censored (*due to pregnancy*) observations as 'never infected', and all censored (*due to pregnancy*) observations as 'immediate failures', at the left and right hand sides of the plots respectively.

Copyright © 2009 John Wiley & Sons, Ltd.
